# Supplementary material for: Comparison of Hodgkin’s Lymphoma in Children and Adolescents. A Twenty Year Experience with MH’96 and LH2004 AIEOP (Italian Association of Pediatric Hematology and Oncology) Protocols
Source: Cancers (Basel). 2020 Jun 18;12(6):1620. doi: 10.3390/cancers12061620 (PMC7352443; doi:10.3390/cancers12061620)
Supplement: Supplementary file 1 [file cancers-12-01620-s001.pdf]

cancers

MDPI

Supplementary Material

Comparison of Hodgkin's Lymphoma in Children and Adolescents. A Twenty Year Experience With MH'96 And LH2004 AIEOP (Italian Association of Pediatric Hematology and Oncology) Protocols.

Roberta Burnelli [1,*], Giulia Fiumana [2], Roberto Rondelli [3], Marta Pillon [4], Alessandra Sala [5], Alberto Garaventa [6], Emanuele S.G. D'Amore [7], Elena Sabattini [8], Salvatore Buffardi [9], Maurizio Bianchi [10], Luciana Vinti [11], Marco Zecca [12], Paola Muggeo [13], Massimo Provenzi [14], Piero Farruggia [15], Francesca Rossi [16], Salvatore D'Amico [17], Elena Facchini [3], Sayla Bernasconi [18], Raffaela De Santis [19], Tommaso Casini [20], Fulvio Porta [21], Irene D'Alba [22], Rosamaria Mura [23], Federico Verzegnassi [24], Antonella Sau [25], Simone Cesaro [26], Katia Perruccio [27], Monica Cellini [2], Patrizia Bertolini [28], Domenico Sperlì [29], Roberta Pericoli [30], Daniela Galimberti [31], Adele Civino [32], Maurizio Mascarin [33] on behalf of the Italian Association of Pediatric Hematology and Oncology.

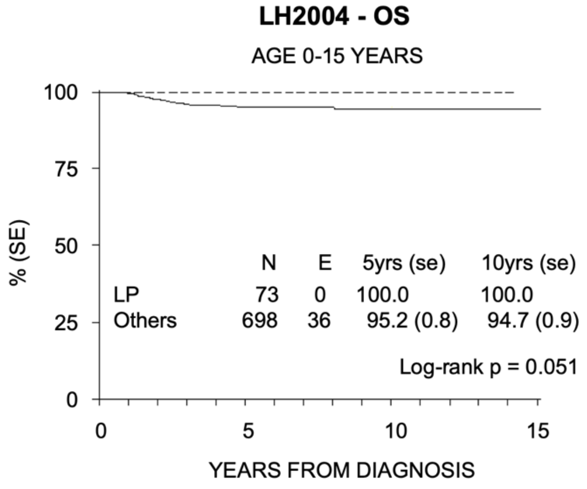

Figure S1. Overall Survival: comparison between nodular Lymphocyte Predominance and other histotypes in children.

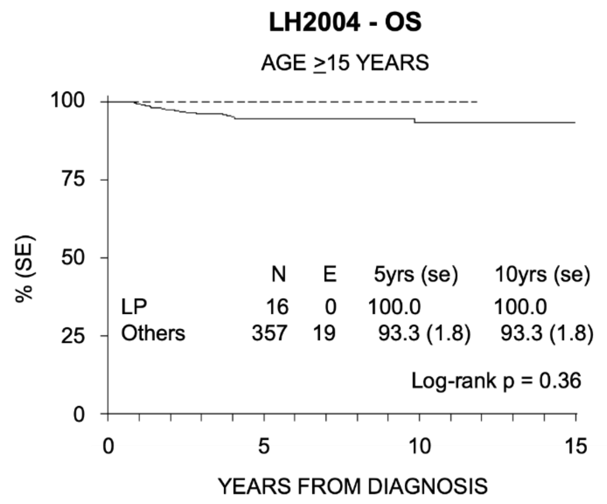

Figure S2. Overall Survival: comparison between nodular Lymphocyte Predominance and other histotypes in adolescents.

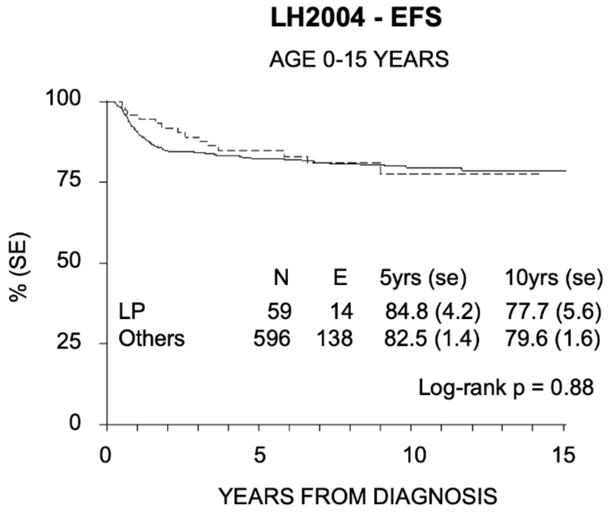

Figure S3. Event-Free Survival: comparison between nodular Lymphocyte Predominance and other histotypes in children.

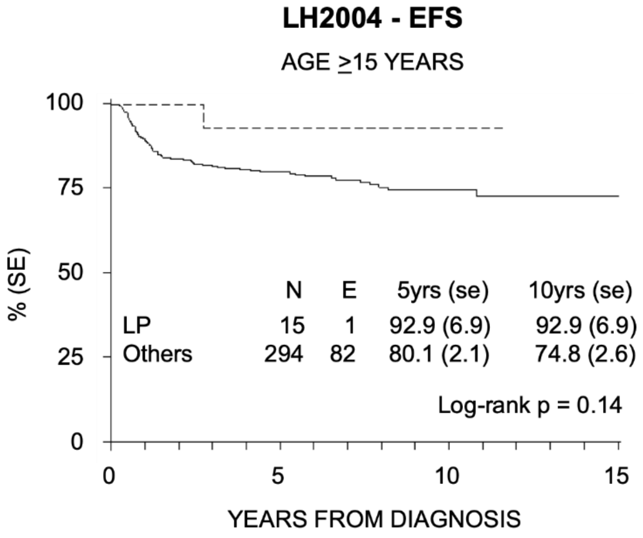

Figure S4. Event-Free Survival: comparison between nodular Lymphocyte Predominance and other histotypes in adolescents.

Table S1. Participating Centers.

1. AOU Citta' Della Salute E Della Scienza Di Torino<br>Presidio Infantile Regina Margherita SC Oncoematologia Pediatrica E Centro Trapianti<br>P.zza Polonia, 94, 10126—Torino
2. IRCCS "Istituto Giannina Gaslini"<br>Via G. Gaslini, 5, 16147—Genova Quarto
3. Fondazione MBBM / AO San Gerardo Clinica Pediatrica Universitaria<br>Via Pergolesi 33, 20900—Monza (Mi)
4. Divisione Pediatria "Mariani"—U.O. di Pediatria—Ospedale "Niguarda Cà Granda"<br>P.zza Ospedale Maggiore, 3, 20162—Milano
5. Fondazione IRCCS, Policlinico San Matteo, SC Oncoematologia Pediatrica<br>Viale Golgi, 19, 27100—Pavia
6. Ospedale Papa Giovanni XXIII USS Oncoematologia Pediatrica<br>Piazza OMS 1, 24100—Bergamo
7. Ospedali Civili, Presidio Ospedale Dei Bambini Oncoematologia Pediatrica E TMO<br>P.Le Spedali Civili,1, 25123—Brescia
8. IRCCS Materno Infantile "Burlo Garofolo"<br>Dipartimento Pediatrico SC Oncoematologia Pediatrica Ed SS Trapianto Di Midollo<br>Via Dell'Istria, 65/1, 34137—Trieste
9. Centro Integrato Di Emato-Oncologia e dell'adolescenza<br>IRCCS Centro Di Riferimento Oncologico—Aviano e A.O.S.Maria degli Angeli – Pordenone<br>Via Franco Gallini 2, 33081—Aviano
10. Azienda Ospedaliera Di Padova Oncoematologia Pediatrica<br>Via Giustiniani, 3, 35128—Padova
11. U.O.C. Oncoematologia Pediatrica, Ospedale Donna Bambino—Azienda Ospedaliera Universitaria Integrata<br>Piazzale Aristide Stefani 1, 37126—Verona
12. Azienda Ospedaliero Universitaria Di Parma, UOC Di Pediatria Ed Oncoematologia<br>Via Gramsci, 14, 43126—Parma
13. Azienda Policlinico Di Modena Pediatria ad Indirizzo oncoematologico<br>Via Del Pozzo, 71, 41100—Modena
14. Policlinico Sant'Orsola Malpighi Clinica Pediatrica Oncologia Ed Ematologia Pediatrica "Lalla Seràgnoli"<br>Via Massarenti, 11, 40138—Bologna
15. Ospedale Infermi U.O. Pediatria SS Oncoematologia Pediatrica<br>Viale Settembrini, 2, 47900—Rimini
16. Azienda Ospedaliero-Universitaria "Anna Meyer"-DAI di Oncoematologia Pediatrica<br>Viale Pieraccini, 24, 50139—Firenze
17. Azienda Ospedaliero Universitaria Senese Policlinico "Le Scotte" Clinica Pediatrica<br>V.le Bracci, 16, 53100—Siena
18. Azienda Ospedaliero-Universitaria Pisana—U.O. Oncoematologia Pediatrica<br>Via Roma 67, 56126—Pisa
19. Azienda Ospedali Riuniti Presidio "G. Salesi", SOS Oncoematologia Pediatrica

Via F. Corridoni, 11, 60123—Ancona
20. Ospedale Civile Dello Spirito Santo—Dipartimento Di Ematologia, Medicina Trasfusionale E Biotecnologie<br>Via Fonte Romana, 8, 65123—Pescara
21. A.O.U. "S.M. Della Misericordia" di Perugia—S.C. Di Oncoematologia Pediatrica con TCSE<br>Piazzale Menghini 1, 06156—S.Andrea Delle Fratte
22. IRCCS Ospedale Pediatrico Bambino Gesù—Dip.to di Oncoematologia e Terapia cellulare e Genica<br>Piazza Sant'Onofrio 4, 00168—Roma
23. Policlinico Umberto I, Universita' "La Sapienza" UOC Oncoematologia Pediatrica<br>Viale Regina Elena 324, 00161—Roma
24. Universita' Degli Studi della Campania "Luigi Vanvitelli"<br>U.O. S.D. Ematologia e Oncologia Pediatrica—DAI Materno Infantile<br>Via Luigi De Crecchio 2—80138—Napoli
25. AORN Santobono-Pausilipon, Dipartimento Di Oncoematologia<br>Via Posillipo, 226, 80123—Napoli
26. IRCCS Ospedale "Casa Sollievo Della Sofferenza", UOC Oncoematologia Pediatrica<br>Viale Cappuccini 7, 71013—San Giovanni Rotondo
27. AO 'Cardinale G.Panico' UO Pediatria<br>Via San Pio X N. 4, 73039—Tricase (Lecce)
28. AOU Policlinico, Dipartimento Di Pediatria<br>Piazza Giulio Cesare, 11, 70124—Bari
29. AO "Bianchi Melacrino Morelli", UOC Ematologia<br>Via Melacrino, 89100—Reggio Calabria
30. A.O. Pugliese-Ciaccio, SOC Ematoncologia Pediatrica<br>Viale Pio X, 88100—Catanzaro
31. SO "Annunziata" UOC Pediatria<br>Via Francesco Migliori , 87100—Cosenza
32. ARNAS Civico Di Cristina E Benfratelli UOC Oncoematologia Pediatrica<br>Piazza Nicola Leotta 4, 90127—Palermo
33. AOU Policlinico Vittorio Emanuele, UOC Ematologia Ed Oncologia Pediatrica Con TMO<br>Via S. Sofia,78, 95123—Catania
34. SC Oncoematologia Pediatrica E Patologia Della Coagulazione,<br>Ospedale Pediatrico Microcitemico "Antonio Cao", Azienda Ospedaliera Brotzu<br>Via Jenner, 09121—Cagliari
35. AOU Sassari, Clinica Pediatrica<br>Viale S.Pietro, 12, 07100—Sassari

Table S2. Ethics Committee approvals.

CENTER	DOCUMENT N°	ETHICS COMMITTEE	APPROVAL DATE	SIGNATURE
ANCONA	18/DG	OSPEDALI RIUNITI DI ANCONA	13/01/2006	D.G. Dr. Paolo Menichetti
AVIANO	Prot. N° 206/D	Comitato Etico del Centro di riferimento oncologico di Aviano	15/02/2005	Presidente Prof. Paolo De Paoli
BARI	2960	Comitato Etico Indipendente della A. O. U. C. Policlinico di Bari	31/05/2006	
BERGAMO	177	Comitato di Bioetica Ospedali Riuniti Bergamo	10/02/2006	
BOLOGNA	1103/2004	Comitato Etico AO di Bologna Policlinico S.Orsola-Malpighi	23/04/2004	Presidente Prof. Ettore Ambrosioni
BRESCIA		Comitato Etico della Azienda Ospedaliera Spedali Civili di Brescia	13/09/2005	Presidente del Comitato Etico Aziendale Prof. Francesco De Ferrari
CAGLIARI	Prot. N° 146/CE/04	Comitato Etico Indipendente Azienda U.S.L. N.8 Cagliari	22/09/2004	Presidente Dr.ssa Nella R. Serci
CATANIA	Verbale N° 68	Comitato Etico AOU Policlinico di Catania	29/07/2005	Segretario Agostino Privitera
CATANZARO		Comitato Etico AO Pugliese-Ciaccio di Catanzaro	13/03/2007	Presidente Dr. Gaetano Muleo
COSENZA	17234	AO di Cosenza Comitato Etico	13/12/2006	Presidente Dr. Osvaldo Perfetti
FIRENZE	Prot. 60	AO Meyer-Firenze Comitato Etico Sperimentazione dei Farmaci	25/07/2005	Presidente Dr.ssa Angela Savelli
GENOVA		Comitato di etica per la ricerca scientifica biomedica, per la buona pratica clinica e per la sperimentazione dei farmaci	15/07/2004	Responsabile della Segreteria scientifica Dr. Ubaldo Rosati
MODENA	Prot n° 144/04	Comitato etico Provinciale di Modena	14/12/2004	Segretario Dr. Saverio Santachiara
MONZA		Comitato Etico AO "San Gerardo" Monza	15/07/2005	Presidente Dr. Vittorio Crespi
NAPOLI SANTOBONO-PAUSILLIPON	Delibera del Direttore Generale N° 422	SSN-AO di rilievo Nazionale "Santobono-Pausillipon"	16.06.2005	Segretario del Comitato Etico Dr. Carlo Maranelli
Napoli II Università	Prot. N° 624 del 16.12.2004	Comitato Etico della Facoltà di Medicina e Chirurgia della II Università degli Studi di Napoli	07.12.2004	Presidente Prof. Luigi Palmieri
PADOVA	codice studio CE 908P	Comitato Etico per la Sperimentazione Clinica della Provincia di Padova	10/04/2006	Segreteria Dr.Camillo Barbisan
PALERMO	Reg. Sper. N° 56/1 del 04/05/2004	Comitato Etico dell'Azienda di Rilievo Nazionale e di Alta Specializzazione Ospedale Civico e Benfratelli, G.Di Cristina e M.Ascoli—Palermo	16/03/2004	Responsabile Amm.ivo dell'ufficio di Segreteria Dr.ssa Silvia Valenti
PARMA	Prot. N° 23219 del 23/09/2004	Comitato Etico Indipendente (IRB/IEC)	04/10/2004	Segretario Dr.ssa Cecilia Morelli
PERUGIA	30184/12/ESS	CEAS-Comitato Etico Aziende Sanitarie Umbria	14/07/2005	
PESCARA	901/CE	AUSL di Pescara Comitato Etico per la Sperimentazione clinica dei armaci	02/08/2005	Presidente Dr. Giuseppe Massimi
PISA	Studio n° 1888/2005	Azienda Ospedaliera Pisana Comitato di Bioetica	07/04/2005	Segretario Franca Cossu
RIMINI	45.05-3.12.6/CE	AUSL di Rimini Commissione Etico-Scientifica	29/04/2005	Presidente Dr. Mario Marzialoni

S.GIOVANNI ROTONDO (FG)		Sezione del Comitato Etico IRCCS Istituto Tumori "Giovanni Paolo II " di Bari presso la Fondazione Casa Sollievo della Sofferenza di San Giovanni Rotondo (FG)	15/10/2005	
SASSARI	Prot. 533/L/2	AUSL n°1 di Sassari Comitato di Bioetica	20/06/2006	
SIENA		AOU Senese Comitato Etico Locale	16/07/2004	Segretario Lucia Polverelli
TORINO	15184/28.3	Commissione Regionale per le Sperimentazioni Cliniche	11/10/2004	Presidente Ing. Gianpiero Cerutti
TRICASE (LE)		Comitato Etico della "Pia Fondazione di Culto e Religione Card. G.Panico" A.O.	06/04/2009	Segretario Amm.vo Dr. Carlo Ingrosso
TRIESTE		Comitato Indipendente per la Bioetica IRCSS-Istituto per l'Infanzia "Burlo Garofalo"-Trieste	16/09/2004	Presidente Furio Bouuqet
VERONA	357/CE	AO Istituti Ospitalieri di Verona Comitato Etico per la Sperimentazione	06/04/2005	Segretaria Dr.ssa Francesca Venturini
